# Supplementary material for: Patterns of ontogenetic evolution across extant marsupials reflect different allometric pathways to ecomorphological diversity
Source: Nat Commun. 2023 May 10;14:2689. doi: 10.1038/s41467-023-38365-0 (PMC10172307; doi:10.1038/s41467-023-38365-0)

Supplementary Information

Patterns of ontogenetic evolution across extant marsupials reflect different allometric pathways to ecomorphological diversity

Laura A. B. Wilson, Camilo López-Aguirre, Michael Archer Suzanne J. Hand, David Flores, Fernando Abdala, Norberto P. Giannini

Contents

Supplementary Table 1. Results of within-group Homogeneity of Slopes comparisons…………………………………………………………………….………….....3

Supplementary Table 2. Results of Homogeneity of Slopes comparisons for pooled ontogenetic trajectories………………………………………………………………………………..……3

Supplementary Table 3. Pairwise comparisons of ontogenetic trajectories across marsupial orders….……………………………………………………………………………….....3

Supplementary Table 4. Pairwise comparisons of ontogenetic trajectories across dietary categories……………………………………………………………………………...….5

Supplementary Table 5. Pairwise comparisons of ontogenetic trajectories for clade * diet interactions..……6

Supplementary Table 6. PC loadings for allometric space………………………………………………………..………………………..…...7

Supplementary Table 7. Morphological disparity for clade and dietary category comparisons……..……..…..8

Supplementary Table 8. Morphological disparity measures across marsupial orders…....………………………………………………………..……………....8

Supplementary Table 9. Morphological disparity for clade * diet interactions…………………………………………...……………....…...…...….....10

Supplementary Table 10. Results of convergence tests among species with similar diet using distance-based indices……………………………………………………………………………….…..11

Supplementary Table 11. Results of convergence tests among species with similar diet using within/between states approach………………..……………...………………………………………………12

Supplementary Table 12. Results of evolutionary model fitting using allometric space coefficients……………………………………………………………………….......13

Supplementary Figure 1. Broken stick model…………………………..………………14

Supplementary Figure 2. Overview of the analysis pipeline…………………...…………..15

Supplementary Figure 3. Allometric space with taxon labels…………………..…………16

Supplementary Table 1. Results of Homogeneity of Slopes (HOS) comparisons. Within groups (dietary habit, partition) results for species ontogenetic allometric trajectories, extracted from cranial measurements of 62 marsupial species (n=2091). Testing was performed using the Bartlett-corrected Likelihood Ratio test against the null (H0) hypothesis of equal slopes among individual species within each group. *P*-values adjusted for multiple comparisons using a Šidák correction.

Comparison	Slope comparison	
	Likelihood ratio	*P*-value
"Ameridelphia"	239.6	2.22e-16
Australidelphia	244.1	2.22e-16
Animalivory	282.9	<2.22e-16
Herbivory	99.48	1.73e-11
Mycophagy	4.857	0.0275
Omnivory	61.12	9.04e-11

Supplementary Table 2. Results of Homogeneity of Slopes (HOS) comparisons. Pooled ontogenetic allometric trajectories results extracted from cranial measurements of 62 marsupial species (n=2091). Testing (two-sided) was performed using the Bartlett-corrected Likelihood Ratio statistic against the null (H0) hypothesis of equal slopes and using the Wald statistic against the null hypothesis of equal elevation (intercept). Partitions: "Ameridelphia" and Australidelphia. *P*-values adjusted for multiple comparisons using a Šidák correction.

Comparison	Slope comparison		Intercept comparison	
	Likelihood ratio	*P*-value	Wald statistic	*P*-value
"Ameridelphia"-Australidelphia	37.43	9.4732e-10	64.24	1.1102e-15
Order	278.1	<2.22e-16	246.0	<2.22e-16
Diet	53.24	1.6298e-11	186.0	<2.22e-16
Partition* Diet	131.7	<2.22e-16	270.4	<2.22e-16

Supplementary Table 3. Results of pair-wise comparisons for pooled ontogenetic allometric trajectories. Comparison results for slope and intercept values across marsupial orders (n=7). Testing (two-sided) was performed using the Bartlett-corrected Likelihood Ratio against the null (H0) hypothesis of equal slopes and using the Wald statistic (1 degree of freedom) against the null hypothesis of equal elevation (intercept). *P*-values were adjusted for multiple comparisons using a Šidák correction.

Comparison		Slope comparison		Intercept comparison	
Order vs	Order	Likelihood ratio	*P*-value	Wald statistic	*P*-value
Dasyuromorphia	Didelphimorphia	38.981	0.000	0.827	1.000
Dasyuromorphia	Diprotodontia	2.303	0.945	13.783	0.004
Dasyuromorphia	Microbiotheria	40.826	0.000	20.123	0.000
Dasyuromorphia	Notoryctemorphia	0.891	1.000	9.206	0.049
Dasyuromorphia	Paucituberculata	180.267	< 2.22e-16	45.253	0.000
Dasyuromorphia	Peramelemorphia	47.872	0.000	3.930	0.640
Didelphimorphia	Diprotodontia	38.777	0.000	60.732	0.000
Didelphimorphia	Microbiotheria	20.086	0.000	58.293	0.000

Didelphimorphia	Notoryctemorphia	0.350	1.000	22.496	0.000
Didelphimorphia	Paucituberculata	113.009	< 2.22e-16	158.156	< 2.22e-16
Didelphimorphia	Peramelemorphia	8.709	0.064	12.499	0.009
Diprotodontia	Microbiotheria	37.052	0.000	55.154	0.000
Diprotodontia	Notoryctemorphia	0.747	1.000	22.356	0.000
Diprotodontia	Paucituberculata	178.505	< 2.22e-16	134.103	< 2.22e-16
Diprotodontia	Peramelemorphia	43.200	0.000	3.188	0.802
Microbiotheria	Notoryctemorphia	0.060	1.000	5.367	0.353
Microbiotheria	Paucituberculata	3.624	0.708	1.376	0.997
Microbiotheria	Peramelemorphia	7.639	0.113	61.709	0.000
Notoryctemorphia	Paucituberculata	0.333	1.000	8.283	0.081
Notoryctemorphia	Peramelemorphia	0.113	1.000	39.276	0.000
Paucituberculata	Peramelemorphia	46.231	0.000	112.291	< 2.22e-16

Supplementary Table 4. Results of pair-wise comparisons for pooled ontogenetic allometric trajectories. Comparison of slope and intercept values across dietary categories (n=4). Testing (two sided) was performed using the Bartlett-corrected Likelihood Ratio against the null (H0) hypotheses of equal slopes and using the Wald statistic (1 degree of freedom) against the null hypothesis of equal elevation (intercept). *P*-values were adjusted for multiple comparisons using a Šidák correction.

Comparison		Slope comparison		Intercept comparison	
Dietary category vs	Dietary category	Likelihood ratio	*P*-value	Wald statistic	*P*-value
Animalivory	Herbivory	7.436	0.038	165.119	< 2.22e-16
Animalivory	Mycophagy	48.894	0.000	32.093	0.000
Animalivory	Omnivory	2.057	0.627	10.133	0.009
Herbivory	Mycophagy	40.857	0.000	0.045	1.000
Herbivory	Omnivory	0.356	0.992	50.054	0.000
Mycophagy	Omnivory	41.621	0.000	39.418	0.000

Supplementary Table 5. Results of pair-wise comparisons for pooled ontogenetic allometric trajectories. Comparison results for slope and intercept values across the interaction between partition ("Ameridelphia", Australidelphia) * diet (n=21). Testing (two-sided) was performed using the Bartlett-corrected Likelihood Ratio against the null (H0) hypotheses of equal slopes and using the Wald statistic (1 degree of freedom) against the null hypothesis of equal elevation (intercept). *P*-values were adjusted for multiple comparisons using a Šidák correction.

Comparison		Slope comparison		Intercept comparison	
Partition*diet vs	Partition*diet	Likelihood ratio	*P*-value	Wald statistic	*P*-value
"Ameridelphia".Animalivory	"Ameridelphia".Herbivory	27.055	0.000	20.110	0.000
"Ameridelphia".Animalivory	"Ameridelphia".Omnivory	15.948	0.001	85.074	< 2.22e-16
"Ameridelphia".Animalivory	Australidelphia.Animalivory	24.422	0.000	15.365	0.002
"Ameridelphia".Animalivory	Australidelphia.Herbivory	1.849	0.982	206.481	< 2.22e-16
"Ameridelphia".Animalivory	Australidelphia.Mycophagy	35.490	0.000	66.434	0.000
"Ameridelphia".Animalivory	Australidelphia.Omnivory	3.347	0.769	2.442	0.929
"Ameridelphia".Herbivory	"Ameridelphia".Omnivory	6.746	0.180	13.717	0.004
"Ameridelphia".Herbivory	Australidelphia.Animalivory	45.275	0.000	1.828	0.983
"Ameridelphia".Herbivory	Australidelphia.Herbivory	31.859	0.000	15.707	0.002
"Ameridelphia".Herbivory	Australidelphia.Mycophagy	0.643	1.000	30.398	0.000
"Ameridelphia".Herbivory	Australidelphia.Omnivory	33.182	0.000	2.937	0.851
"Ameridelphia".Omnivory	Australidelphia.Animalivory	47.056	0.000	18.968	0.000
"Ameridelphia".Omnivory	Australidelphia.Herbivory	23.704	0.000	7.856	0.101
"Ameridelphia".Omnivory	Australidelphia.Mycophagy	12.006	0.011	11.282	0.016
"Ameridelphia".Omnivory	Australidelphia.Omnivory	23.288	0.000	24.794	0.000
Australidelphia.Animalivory	Australidelphia.Herbivory	16.438	0.001	63.300	0.000
Australidelphia.Animalivory	Australidelphia.Mycophagy	54.431	0.000	13.842	0.004
Australidelphia.Animalivory	Australidelphia.Omnivory	3.998	0.624	1.622	0.991
Australidelphia.Herbivory	Australidelphia.Mycophagy	40.581	0.000	0.254	1.000
Australidelphia.Herbivory	Australidelphia.Omnivory	0.861	1.000	65.313	0.000
Australidelphia.Mycophagy	Australidelphia.Omnivory	41.825	0.000	47.143	0.000

Supplementary Table 6. PC loadings extracted from allometric space. Allometric space constructed using ontogenetic allometric trajectories of cranial measurements for 50 species of marsupials (n=1949). Abbreviations: BB, breadth of braincase; BPAL, breadth of palate; CIL, condyle-incisive length; HD, height of mandibular body; HM, height of muzzle; LC, length of coronoid process; LD, length of dentary; LN, length of nasals; LPAL, length of palate; LPos, length of lower postcanine row; OH, height of occipital plate; ORB, length of orbit; PAL, length of palate; UPos, length of upper postcanine row; ZB, zygomatic breadth.

Trait	PC1	PC2	PC3	PC4	PC5	PC6	PC7	PC8	PC9	PC10	PC11	PC12	PC13	PC14
CIL	-0.190	0.270	-0.532	0.181	0.287	-0.007	0.236	-0.267	-0.164	0.022	0.398	0.174	0.049	-0.383
ZB	-0.332	0.106	0.239	-0.225	0.169	0.169	0.135	0.055	-0.084	-0.062	-0.584	0.410	0.062	-0.411
BB	-0.237	-0.164	-0.415	-0.114	-0.523	0.061	-0.019	0.158	0.062	-0.541	-0.056	-0.108	0.324	-0.116
OH	-0.289	-0.042	0.329	0.099	-0.266	-0.277	0.028	-0.139	-0.732	0.111	0.108	-0.107	0.237	0.037
ORB	-0.075	-0.539	-0.148	-0.181	0.364	-0.082	0.609	0.029	-0.129	-0.097	-0.100	-0.151	-0.064	0.276
LN	-0.295	-0.264	0.029	-0.202	-0.082	0.398	-0.016	0.445	0.056	0.494	0.411	0.100	0.084	-0.075
LPAL	0.127	-0.450	0.300	0.449	-0.237	0.203	0.218	-0.341	0.195	-0.131	0.153	0.350	-0.007	-0.168
BPAL	-0.257	-0.103	-0.242	0.665	-0.001	0.123	-0.069	0.124	0.013	0.303	-0.439	-0.307	-0.089	-0.023
Upos	-0.287	0.310	0.260	-0.013	0.043	0.110	0.305	-0.273	0.446	0.077	0.041	-0.343	0.471	0.163
LD	-0.296	-0.053	0.140	0.105	-0.016	-0.744	0.073	0.294	0.376	-0.006	0.129	0.074	-0.161	-0.209
HD	-0.366	0.157	-0.098	0.171	0.012	0.026	-0.083	0.033	0.016	-0.129	0.024	0.542	-0.044	0.694
Lpos	-0.227	-0.304	0.156	0.077	0.553	0.056	-0.564	-0.069	0.009	-0.328	0.153	-0.139	0.195	-0.070
HM	-0.341	0.172	0.200	-0.047	-0.104	0.284	0.088	-0.052	-0.052	-0.304	0.175	-0.305	-0.699	-0.043
HC	-0.260	-0.263	-0.217	-0.358	-0.173	-0.141	-0.267	-0.618	0.160	0.324	-0.132	0.025	-0.193	0.015

Supplementary Table 7. Morphological disparity (Procrustes Variance) measures. Results reported for partition (“Ameridelphia”, Australidelphia) and dietary (grey shading) category comparisons. Procrustes Variance values were calculated using principal component (PC) scores extracted from allometric space.

Pairwise difference/*P*-value	“Ameridelphia”	Australidelphia	Animalivory	Herbivory	Mycophagy	Omnivory
“Ameridelphia”		0.775				
Australidelphia	2.750					
Animalivory				0.352	0.090	0.334
Herbivory			8.001		0.058	0.831
Mycophagy			30.540	38.541		0.061
Omnivory			10.635	2.634	41.175	
Procrustes Variance	10.234	12.984	15.364	7.363	45.904	4.729

Supplementary Table 8. Morphological disparity (Procrustes Variance) measures for Order comparisons. Procrustes Variance values were calculated using principal component (PC) scores extracted from allometric space. Absolute pairwise differences in Procrustes variance are test statistics (one-sided), and are shown below the diagonal. The statistical significance of pairwise distances between groups was assessed using a permutation test (10,000 iterations), with the corresponding *P*-value shown above the diagonal. Procrustes Variance for each Order shown at the bottom of the table, shaded grey.

Pairwise difference/*P*-value	Dasyuromorphia	Didelphimorphia	Diprotodontia	Microbiotheria	Paucituberculata	Peramelemorphia
Dasyuromorphia		0.4012	0.2709	0.1974	0.1974	0.2881
Didelphimorphia	9.204		0.9147	0.5048	0.4798	0.6681
Diprotodontia	10.389	1.184		0.6284	0.577	0.8113
Microbiotheria	17.054	7.850	6.666		0.8929	0.6363
Paucituberculata	17.750	8.546	7.362	0.696		0.5857
Peramelemorphia	13.953	4.750	3.566	3.100	3.796	
Procrustes Variance	20.753	11.549	10.365	3.699	3.003	6.800

Supplementary Table 9. Morphological disparity (Procrustes Variance) measures for partition (i.e., "Ameridelphia", Australidelphia)* diet comparisons. Procrustes Variance values were calculated using principal component (PC) scores extracted from allometric space. Absolute pairwise differences in Procrustes variance are test statistics (one-sided), and are shown below the diagonal. The statistical significance of pairwise distances between groups was assessed using a permutation test (10,000 iterations), with the corresponding *P*-value shown above the diagonal. Procrustes Variance for each partition * diet interaction shown at the bottom of the table, shaded grey.

Pairwise difference/*P*-value	"Ameridelphia". Animalivory	"Ameridelphia". Herbivory	"Ameridelphia". Omnivory	Australidelphia. Animalivory	Australidelphia. Herbivory	Australidelphia. Mycophagy	Australidelphia. Omnivory
"Ameridelphia".Animalivory		0.822	0.528	0.529	0.698	0.074	0.575
"Ameridelphia".Herbivory	2.440		0.484	0.444	0.915	0.134	0.584
"Ameridelphia".Omnivory	7.255	4.816		0.273	0.827	0.119	0.913
Australidelphia.Animalivory	6.769	9.209	14.025		0.248	0.101	0.299
Australidelphia.Herbivory	4.272	1.832	2.984	11.041		0.058	0.862
Australidelphia.Mycophagy	34.366	36.806	41.621	27.597	38.638		0.077
Australidelphia.Omnivory	6.586	4.146	0.669	13.355	2.314	40.952	
Procrustes Variance	11.538	9.098	4.283	18.308	7.267	45.904	4.952

Supplementary Table 10. Results of convergence tests among representatives of Ameridelphian and Australidelphian marsupials. Convergence was assessed based on four distance-based statistics (C1-C4), using principal component (PC) scores extracted from allometric space, constructed from cranial measurements taken on ontogenetic series of 50 species. C statistic values (one-sided) measure convergence on an increasing scale, such that zero reflects an absence of convergence and larger values denote greater convergence. C1, ranging between 0 and 1, measures the phenotypic distance between convergent tips relative to the maximum evolutionary distance between two lineages, reflecting how much subsequent evolution has reduced inter-lineage distance. The C2 statistic assesses the magnitude of evolution that has occurred as a result of convergence, with larger values indicating greater amounts of convergence. Similar to C1, C3 and C4 test statistics reflect proportions such that C3 indicates how much convergence has occurred as a proportion of the total amount of evolution along lineages leading from their common ancestor and C4 indicates how much convergence has occurred relative to the entire clade, defined by the common ancestor of convergent taxa. Significance, returned as a *P*-value was assessed using simulated evolution of the observed data under Brownian motion (300 simulation runs).

Dietary category	Convergence index (C)	Value	*P*-value
Animalivory	C1	0.182	0.010
	C2	0.631	0.037
	C3	0.084	0.023
	C4	0.005	0.395
Herbivory	C1	0.255	0.003
	C2	0.818	0.000
	C3	0.117	0.007
	C4	0.007	0.053
Omnivory	C1	0.045	0.897
	C2	0.114	0.963
	C3	0.016	0.957
	C4	0.001	0.987

Supplementary Table 11. Results of convergence tests among representatives of Ameridelphian and Australidelphian marsupials. Convergence tests were run using allometric coefficients (species-level regression of shape variables ~ size) and principal component (PC) scores extracted from allometric space, constructed from cranial measurements taken on ontogenetic series of 50 species. The convergence test (one-sided) adopted a within/between states approach, outputting the test statistic of mean angle between all possible species pairs (i.e., Ameridelphian animalivore – Australidelphian animalivore, Ameridelphian omnivore – Australidelphian omnivore) (ang.state) plus the test statistic of mean angle divided by time distance (ang.state.time statistic). Significance of these two test statistics was evaluated by comparing the results to 300 randomly generated angles computed by species extracted by chance from the observed tree. The dietary categories of herbivory and mycophagy were excluded from this analysis due to being represented either in only one partition (mycophagy) or by only one species per partition (herbivory).

Dataset	Dietary category	ang.state (degrees)	ang.state.time	*P*-value (ang.state)	*P*-value (ang.state.time)
Allometric coefficients	Animalivory	72.432	0.430	0.372	0.001
	Omnivory	65.435	0.393	0.174	0.007
Allometric space PC scores	Animalivory	86.653	0.515	0.03	0.001
	Omnivory	92.296	0.554	0.625	0.03

Supplementary Table 12. Results of evolutionary modelling fitting. Modelling was undertaken using the first four Principal Components (PC) of allometric space. Partitions: "Ameridelphia" and Australidelphia. Models were grouped into three broad categories: Early Burst (EB), Brownian Motion (BM) and Ornstein-Uhlenbeck (OU). Seven different BM models were fit: a single-rate BM model (BM1), a multiple-rate BM model based on partitions (BMMs), a multiple-rate BM model based on diet (BMMd), a multiple-rate BM model based on a partition*diet interaction (BMMsd), a BM model with multiple ancestral states based on partition (BMMsm), a BM model with multiple ancestral states based on diet (BMMdm) and a BM model with multiple ancestral states based on a partition*diet interaction (BMMsdm). Additionally, four OU models were assessed, one assuming a single optimum (OU1), one assuming different optima based on partition (OUs), one assuming different optima based on diet (OUd), and one assuming different optima based on a partition*diet interaction (OUsd). Model selection was based on sample-size corrected Akaike information criteria (AICc), with models with a delta (d) AICc below two considered as the best-supported models.

Model	LogLike	AICc	dAICc
BM	-392.079	814.429	78.553
BMMs	-359.575	774.007	38.131
BMMd	-346.689	806.926	71.050
BMMsd	-298.897	834.593	98.718
BMMsm	-359.568	784.634	48.758
BMMdm	-335.060	826.764	90.888
BMMsdm	-287.189	962.497	226.621
EB	-392.079	816.768	80.892
OU1	-340.509	735.876	0
OUs	-339.682	744.861	8.985
OUd	-326.534	741.412	5.537
Ousd	-322.2611	771.6745	35.7988

Supplementary Figure 1. Results of a broken stick model applied to the principal components of allometric space. A broken stick model was implemented to assess the significance of variance accounted for by each axis. Eigenvalues are shown in the top panel, and their corresponding (observed) percentage of variance is shown in the bottom panel as compared to the expected proportions of variance (red bars) that result from a broken stick model. Source data are provided as a Source Data file.

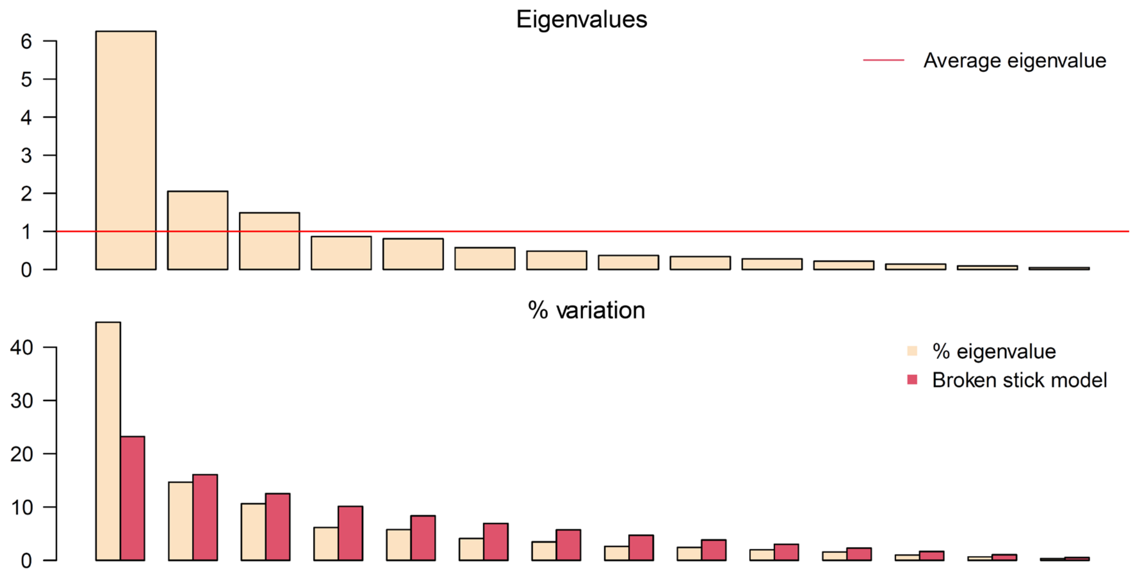

Supplementary Figure 2. Overview of data analysis pipeline. See Methods for full descriptions of each step.

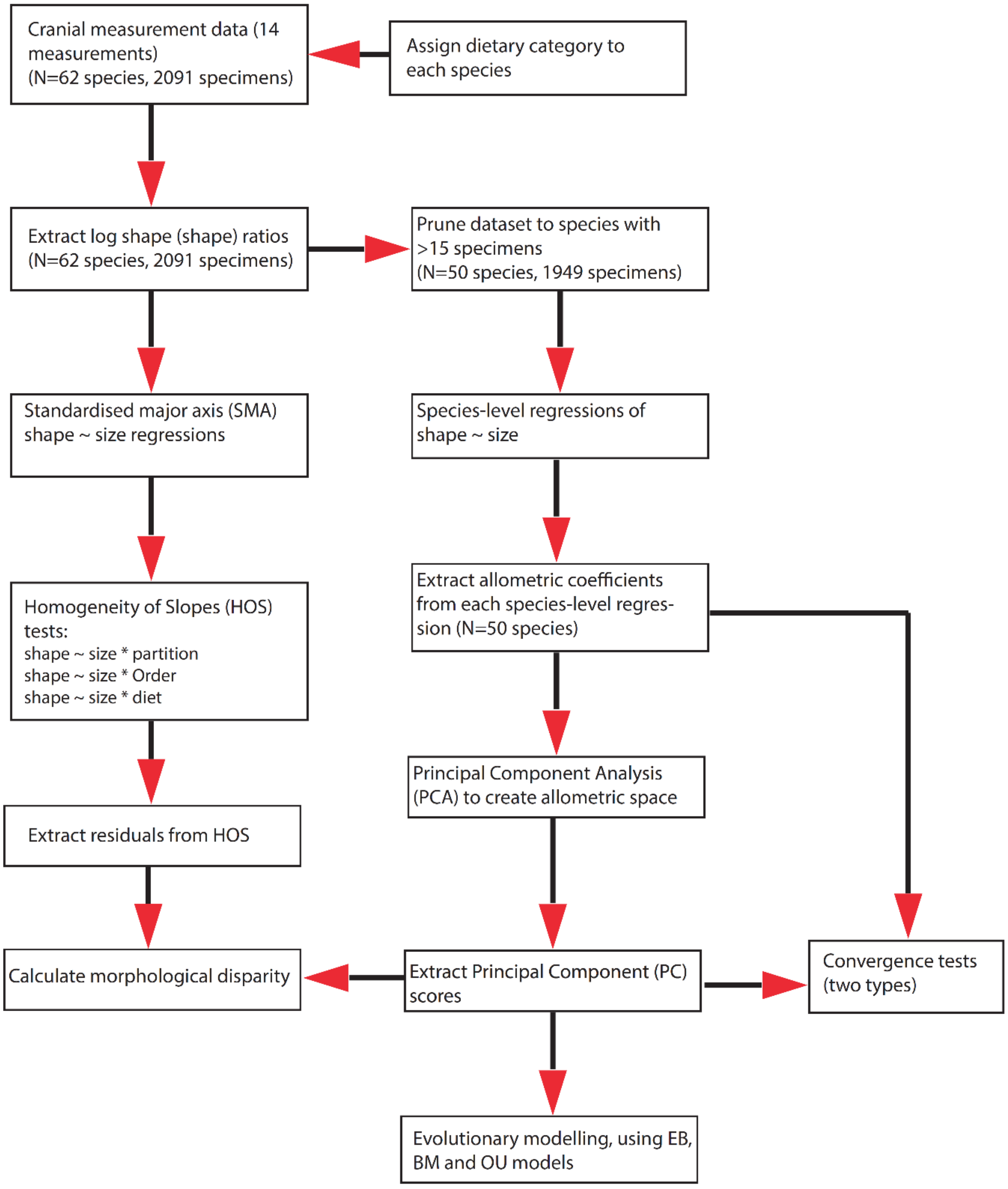

Supplementary Figure 3. Allometric space with taxonomic shorthand labels. Shorthands adopt the first three letters of the genus name and the first three letters of the species epithet (e.g., *Dasyurus maculatus* = dasmac). See main text Figure 1 for species examined in this study. Ellipses are coloured by dietary habit: animalivory (red), herbivory (green), omnivory (purple), mycophagy (blue). Source data are provided as a Source Data file.

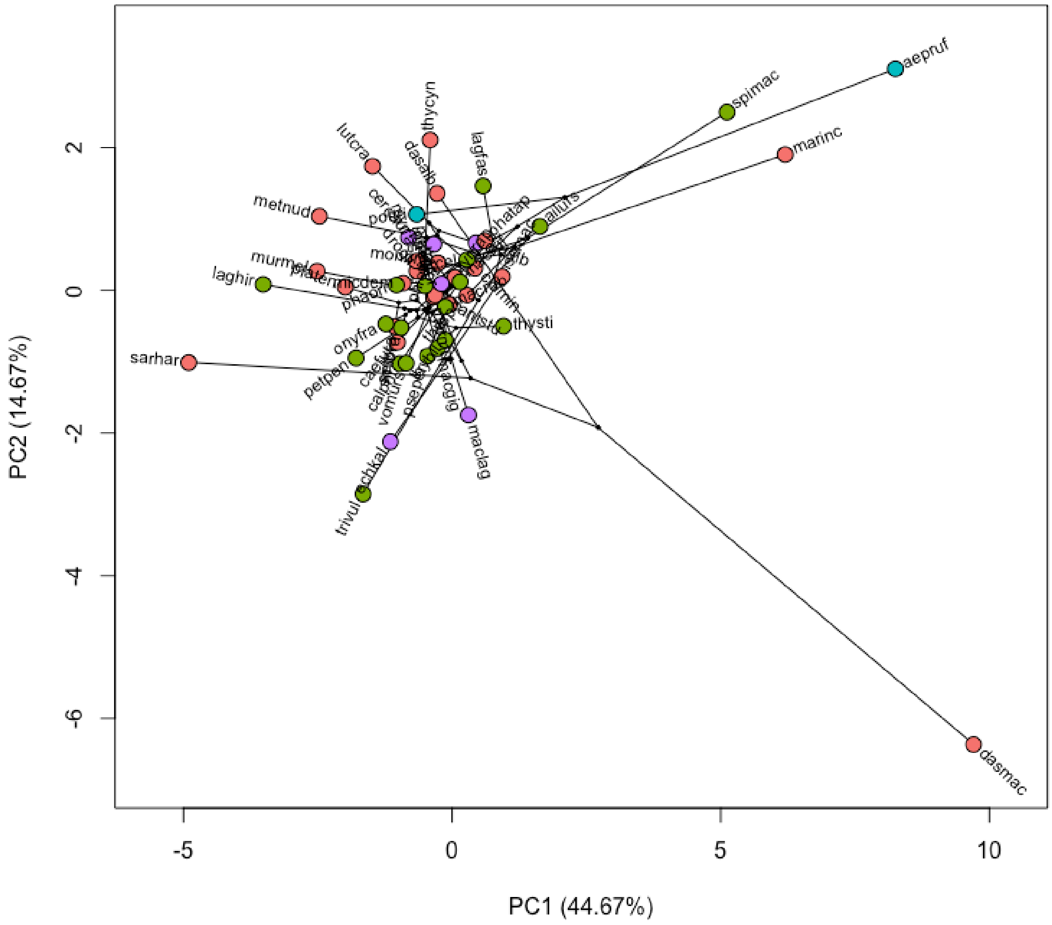

Supplement: Supplementary file 1 — Supplementary Information [file 41467_2023_38365_MOESM1_ESM.pdf]
