# Supplementary material for: Patterns of ontogenetic evolution across extant marsupials reflect different allometric pathways to ecomorphological diversity
Source: Nat Commun. 2023 May 10;14:2689. doi: 10.1038/s41467-023-38365-0 (PMC10172307; doi:10.1038/s41467-023-38365-0)
Supplement: Supplementary file 3 — Reporting Summary [file 41467_2023_38365_MOESM3_ESM.pdf]

nature portfolio

Corresponding author(s): Laura Wilson

Last updated by author(s): Apr 5, 2023

Reporting Summary

Nature Portfolio wishes to improve the reproducibility of the work that we publish. This form provides structure for consistency and transparency in reporting. For further information on Nature Portfolio policies, see our Editorial Policies and the Editorial Policy Checklist.

Statistics

For all statistical analyses, confirm that the following items are present in the figure legend, table legend, main text, or Methods section.

n/a	Confirmed	
☐	☒	The exact sample size (*n*) for each experimental group/condition, given as a discrete number and unit of measurement
☐	☒	A statement on whether measurements were taken from distinct samples or whether the same sample was measured repeatedly
☐	☒	The statistical test(s) used AND whether they are one- or two-sided<br>*Only common tests should be described solely by name; describe more complex techniques in the Methods section.*
☐	☒	A description of all covariates tested
☐	☒	A description of any assumptions or corrections, such as tests of normality and adjustment for multiple comparisons
☐	☒	A full description of the statistical parameters including central tendency (e.g. means) or other basic estimates (e.g. regression coefficient) AND variation (e.g. standard deviation) or associated estimates of uncertainty (e.g. confidence intervals)
☐	☒	For null hypothesis testing, the test statistic (e.g. *F*, *t*, *r*) with confidence intervals, effect sizes, degrees of freedom and *P* value noted<br>*Give P values as exact values whenever suitable.*
☒	☐	For Bayesian analysis, information on the choice of priors and Markov chain Monte Carlo settings
☒	☐	For hierarchical and complex designs, identification of the appropriate level for tests and full reporting of outcomes
☒	☐	Estimates of effect sizes (e.g. Cohen's *d*, Pearson's *r*), indicating how they were calculated

Our web collection on statistics for biologists contains articles on many of the points above.

Software and code

Policy information about availability of computer code

Data collection	No software was used for data collection.
Data analysis	All data procedures, along with statistical analyses, were conducted in the open source R environment v. 4.1.3. The R code is deposited in Github and can be accessed via this link: https://github.com/labw09/marsupialsallom<br>The R packages used in this research were: geomorph (version 4.04), convevol (version 1.3), phytools (version 1.2-0), RRphylo (version 2.5.8), mvMORPH (version 1.1.6), smatr (version 3.4-8), RColorBrewer (version 1.1-3)

For manuscripts utilizing custom algorithms or software that are central to the research but not yet described in published literature, software must be made available to editors and reviewers. We strongly encourage code deposition in a community repository (e.g. GitHub). See the Nature Portfolio guidelines for submitting code & software for further information.

Data

Policy information about availability of data

All manuscripts must include a data availability statement. This statement should provide the following information, where applicable:

- Accession codes, unique identifiers, or web links for publicly available datasets
- A description of any restrictions on data availability
- For clinical datasets or third party data, please ensure that the statement adheres to our policy

The data generated in this study have been deposited in the Zenodo database at https://zenodo.org/record/7804164#.ZC5No_ZBxZc. The data generated in this

study are provided in the Source Data file. Source data are provided as a Source Data file. Data were compiled from Flores et al (2018, 2022) and are available at https://link.springer.com/article/10.1007/s13127-018-0369-3#Sec16 and https://link.springer.com/referenceworkentry/10.1007/978-3-030-88800-8_6-1#Sec18
Phylopic images are: https://www.phylopic.org/images/55e1c3e9-940c-486a-90d7-4b8e5663d248/thylacinus-cynocephalus, https://www.phylopic.org/images/73e9af73-e873-4aa8-90be-8d4a9afb8617/perameles-bougainville, Sarah Werning (unchanged) https://www.phylopic.org/images/dde4f926-c04c-47ef-a337-927ceb36e7ef/dromiciops-gliroides, https://www.phylopic.org/images/0e1fe113-feb7-46db-8a10-12f22f80332c/didelphis-virginiana, Sarah Werning (unchanged) https://www.phylopic.org/images/f34ca418-a0d9-4ed7-bc3c-a74bcdeae443/caenolestes-fuliginosus, https://www.phylopic.org/images/b62bab6e-99e9-4525-9b89-f5fb94742112/macropus-macropus.

Human research participants

Policy information about studies involving human research participants and Sex and Gender in Research.

Reporting on sex and gender	not applicable
Population characteristics	not applicable
Recruitment	not applicable
Ethics oversight	not applicable

Note that full information on the approval of the study protocol must also be provided in the manuscript.

Field-specific reporting

Please select the one below that is the best fit for your research. If you are not sure, read the appropriate sections before making your selection.

☐ Life sciences ☐ Behavioural & social sciences ☒ Ecological, evolutionary & environmental sciences

For a reference copy of the document with all sections, see nature.com/documents/nr-reporting-summary-flat.pdf

Ecological, evolutionary & environmental sciences study design

All studies must disclose on these points even when the disclosure is negative.

Study description	Macroevolutionary analyses of ontogenetic allometry of the cranium in extant marsupials, using a suite of analyses that account for phylogenetic relationships (phylogenetic comparative methods) and that quantify the amount of variation (disparity) and the extent of morphological convergence in patterns of ontogenetic allometry. The applied models to assess ontogenetic allometry considered the null hypothesis of equal slopes and intercepts (shape ~ size). This null was compared against models with the interaction term species (shape ~ size * species) (representing species-level testing of slopes, un-pooled data), followed by the interaction terms: partition ("Ameridelphia", Australidelphia) (shape ~ size*partition), Order (shape ~ size*Order), and diet (shape ~ size*diet). The latter list represent pooled trajectory models. P values were adjusted for multiple comparisons using a Sidak correction. Assessment of fit for evolutionary models was undertaken using sample-size corrected Akaike information criteria (AICc). Models with a delta AICc below 2.0 were considered as the best supported models. The covariate of 'diet' was assigned using the most comprehensive assessment of marsupial diet published to date, capturing data for 193 species of extant marsupials. These data are available as a supplement to the publication: Amador, L. I. & Giannini, N. P. Evolution of diet in extant marsupials: emergent patterns from a broad phylogenetic perspective. Mammal Review 51, 178-192 (2020).
Research sample	The sample comprised 2091 specimens representing ontogenetic series of 62 species, comprising coverage of 18 families of marsupials including members of all seven living Orders. Raw ontogenetic measurement data (ontogenetic series) were collected and compiled from published data from Flores, D. A., Giannini, N. & Abdala, F. Evolution of post-weaning skull ontogeny in New World opossums (Didelphidae). Organisms Diversity & Evolution 18, 367-382 (2018) and from Flores D, Abdala F, Giannini N. Postweaning skull growth in living American and Australasian marsupials: allometry and evolution. In: Cáceres NC, Dickman CR, editors. American and Australasian Marsupials: An Evolutionary, Biogeographical, and Ecological Approach: Springer Cham (2022). The study sample was composed to maximise representation of ecomorphological and taxonomic diversity across American and Australasian marsupials, targeting species representations for animalivorous, herbivorous, mycophagous and ominivorous categories, as well as body mass across five orders of magnitude, reflecting extant diversity across marsupials. Species selection was finalised by targeting species with high representation (>15) of ontogenetic samples within accessioned collections. Among the more speciose groups, body mass ranges were also targeted to maximise representation across the spectrum of extant adult body mass (4 g to 16.7 kg in Dasyuromorphia, from 19 g to 1.3 kg in Didelphimorphia and from 9 g to 39 kg in Diprotodontia).<br>Measurements were recorded in millimeters (mm) using callipers in person at Natural History Museums in the US, Brazil, and Australia.
Sampling strategy	Natural History Museums with the largest collections of dry skull material for marsupials were targeted in North and South America and Australia, to capture species that represent the entire diversity of extant marsupials. Multiple collections were chosen to maximise sampling of juvenile specimens, which are comparatively rare and incomplete in collections of skeletal material (e.g., 1-2 specimens), compared to adult individuals. Sampled ontogenetic trajectories targeted a minimum of 15 specimens, i.e., a greater number of specimens than shape ratio variables (n=14), to avoid 'large p, small n' situation whereby the number of variables (p) exceeds the number of specimens (n).

	Cranial measurement data were collected from institutions in North and South America, and Australia, as follows: Australian Museum, Sydney (AM), American Museum of Natural History, New York (AMNH), Centro Nacional Patagónico, Puerto Madryn (CNP), Field Museum of Natural History, Chicago (FMNH), Museo Argentino de Ciencias Naturales Bernando Rivadavia, Buenos Aires (MACN), Museo de La Plata, La Plata (MLP), Museu Nacional Universidade Federal do Rio de Janeiro, Rio De Janeiro (MNRIO), Museu de Zoologia da Universidade de São Paulo, São Paulo (MZUSP), and Western Australian Museum,
Data collection	A series of linear measurements, comprising both neurocranial and splanchnocranial variables and accommodating all the major dimensions of the skull were recorded on each specimen. These were: BB, breadth of braincase; BPAL, breadth of palate; CBL, condylobasal length; HD, height of mandibular body; HM, height of muzzle; LC, length of coronoid process; LD, length of dentary; LN, length of nasals; LPAL, length of palate; LPos, length of lower postcanine row; OH, height of occipital plate; ORB, length of orbit; PAL, length of palate; UPos, length of upper postcanine row; ZB, zygomatic breadth. Measurement data were collected at natural history museum collections in person, involving collection of the raw data by NPG, FA, DF and LABW. All authors used the same measurement collection scheme, as was also followed for the published data that were added to the data set (Flores et al., 2018, 2022). Data were collected using digital callipers on dry skull material housed in museum institutions (natural history/zoology collections).
Timing and spatial scale	Data collection occurred during several museum visits on different continents, over the course of 2017, 2018 and 2019 (Jan-May). Data collection was spread out over several years due to teaching and research commitments of the authors, and availability of access to national collections. Data matrices were collated Aug/Sept 2019, however further research was halted due to the pandemic. As these data were collected from historically-stored specimens (or collated from published sources), the timing of data collection did not impact the data results (i.e., compared to e.g., fresh tissue or experimental set-up). Data analyses commenced in June 2022 and were completed in November 2022, additional data presentation was undertaken in February 2023. Spatial scale of the data comprised representatives from the disjunct geographic areas where extant marsupials occur - Australasia and North and South America, only. In terms of temporal scale, the study did not include fossil diversity because the aim was to quantify ontogenetic allometry, which required complete juvenile/ontogenetic material (i.e., undamaged dry skull bones) in moderate sample sizes (n=15), which was not possible for most extinct representatives of Marsupialia and stem relatives.
Data exclusions	Several specimens was excluded due to a gross error in measurement, representing an order magnitude incorrect recording of measurements. Only specimens with a complete set of measurements for all variables were included.
Reproducibility	Experiments were not conducted in this study. All code is provided to reproduce results presented in this study. All R package versions are listed.
Randomization	Our data are species data, meaning that species are not statistically independent due to shared evolutionary history. Each species was assigned to groupings according to its taxonomy ('Superorder', Order), which was based on the maximum likelihood phylogeny of Mitchell et al., (2014) that comprises 97% of extant marsupial diversity at genus level and 58% at species level, and locally adjusted branch lengths for interrelationships within Caenolestidae, extracted from Ojala-Barbour et al., (2013) and for didelphids, taken from Amador and Giannini (2016). Species were also grouped according to dietary habit. This grouping was created following the comprehensive four-state dietary scheme created by Amador and Giannini (2016), represented as animalivory, herbivory, mycophagy and omnivory. This classification system was created by procuring information from the literature on natural diet, using a minimum of five reference sources per taxon, and assignments for the four-state scheme were based on predominant food source in the diet (> 50% frequency of consumption).
Blinding	Blinding was not undertaken as this study involved the collection of raw data from natural history museum collections, requiring confirmation of taxonomic assignment for each individual included in the study and anatomical/morphological knowledge of the cranium in marsupial mammals.

Did the study involve field work? ☐ Yes ☒ No

Reporting for specific materials, systems and methods

We require information from authors about some types of materials, experimental systems and methods used in many studies. Here, indicate whether each material, system or method listed is relevant to your study. If you are not sure if a list item applies to your research, read the appropriate section before selecting a response.

Materials & experimental systems

n/a	Involved in the study
☒	☐ Antibodies
☒	☐ Eukaryotic cell lines
☒	☐ Palaeontology and archaeology
☒	☐ Animals and other organisms
☒	☐ Clinical data
☒	☐ Dual use research of concern

Methods

n/a	Involved in the study
☒	☐ ChIP-seq
☒	☐ Flow cytometry
☒	☐ MRI-based neuroimaging
